# Blood circulating bacterial DNA in hospitalized old COVID-19 patients

**DOI:** 10.1186/s12979-023-00401-4

**Published:** 2023-12-18

**Authors:** Robertina Giacconi, Patrizia D’Aquila, Maurizio Cardelli, Francesco Piacenza, Elisa Pierpaoli, Giada Sena, Mirko Di Rosa, Anna Rita Bonfigli, Roberta Galeazzi, Antonio Cherubini, Massimiliano Fedecostante, Riccardo Sarzani, Chiara Di Pentima, Piero Giordano, Roberto Antonicelli, Fabrizia Lattanzio, Giuseppe Passarino, Mauro Provinciali, Dina Bellizzi

**Affiliations:** 1 Advanced Technology Center for Aging Research, IRCCS INRCA, Ancona, Italy; 2 Department of Biology, Ecology and Earth Sciences, University of Calabria, 87036 Rende, Italy; 3 Unit of Geriatric Pharmacoepidemiology and Biostatistics, IRCCS INRCA, Ancona, Italy; 4 Scientific Direction, IRCCS INRCA, Ancona, Italy; 5 Clinical Laboratory and Molecular Diagnostic, Italian National Research Center On Aging, IRCCS INRCA, Ancona, Italy; 6 Geriatria, Accettazione Geriatrica e Centro Di Ricerca Per L’invecchiamento, IRCCS INRCA, Ancona, Italy; 7 Department of Clinical and Molecular Sciences, Università Politecnica Delle Marche, Ancona, Italy, Internal Medicine and Geriatrics, Italian National Research Centre On Aging, Hospital “U. Sestilli”, IRCCS INRCA, Ancona, Italy; 8 Cardiology Unit, IRCCS INRCA, Ancona, Italy

**Keywords:** COVID-19, Hospitalization, Circulating bacterial DNA, Aging, Inflammation, Mortality

## Abstract

**Background:**

Coronavirus disease COVID-19 is a heterogeneous condition caused by SARS-CoV-2 infection. Generally, it is characterized by interstitial pneumonia that can lead to impaired gas-exchange, acute respiratory failure, and death, although a complex disorder of multi-organ dysfunction has also been described. The pathogenesis is complex, and a variable combination of factors has been described in critically ill patients. COVID-19 is a particular risk for older persons, particularly those with frailty and comorbidities. Blood bacterial DNA has been reported in both physiological and pathological conditions and has been associated with some haematological and laboratory parameters but, to date, no study has characterized it in hospitalized old COVID-19 patients The present study aimed to establish an association between blood bacterial DNA (BB-DNA) and clinical severity in old COVID-19 patients.

**Results:**

BB-DNA levels were determined, by quantitative real-time PCRs targeting the 16S rRNA gene, in 149 hospitalized older patients (age range 65–99 years) with COVID-19. Clinical data, including symptoms and signs of infection, frailty status, and comorbidities, were assessed. BB-DNA was increased in deceased patients compared to discharged ones, and Cox regression analysis confirmed an association between BB-DNA and in-hospital mortality. Furthermore, BB-DNA was positively associated with the neutrophil count and negatively associated with plasma IFN-alpha. Additionally, BB-DNA was associated with diabetes.

**Conclusions:**

The association of BB-DNA with mortality, immune-inflammatory parameters and diabetes in hospitalized COVID-19 patients suggests its potential role as a biomarker of unfavourable outcomes of the disease, thus it could be proposed as a novel prognostic marker in the assessment of acute COVID-19 disease.

**Supplementary Information:**

The online version contains supplementary material available at 10.1186/s12979-023-00401-4.

## Background

Coronavirus disease (COVID-19) is an illness caused by SARS-CoV-2 infection that affects multiple organ systems and is characterized by a spectrum of clinical manifestations ranging from asymptomatic infections to severe disease resulting in mortality [1]. The heterogeneity of the disease, in terms of severity and progression, is strictly linked to host factors, including age, male sex, increased BMI, frailty, and pre-existing comorbid chronic conditions [2–7]. Ethnicity is also relevant to COVID-19 susceptibility and severity. Host genetic predisposition to COVID-19 was reported, and whole genome and candidate gene association studies have been performed. Demographic evidence from around the worldwide suggests that age is the most significant risk factor for severe COVID-19 disease since severe clinical manifestations and mortality in COVID-19 patients increase among older subjects. It was reported that the physiological decline occurring with age, accompanied by progressive biological changes in the immune system, frailty, and age-related comorbidities, renders older individuals more prone to adverse outcomes of SARS-CoV-2 infection [8–10]. Moreover, in patients with severe COVID-19, acute respiratory distress syndrome (ARDS), multiple organ dysfunction syndrome and mortality can result from cytokine storm, caused by the excessive and uncontrolled release of pro-inflammatory cytokines. Several studies have demonstrated that IL-6, IL-10 and TNF-α play a significant role in the inflammatory storm, through complex inflammatory networks and may lead to DNA damage and genome instability due to the diminished DNA repair capacity [11–13]. Type I interferons (IFNs), predominantly comprised of IFN-α and IFN-β, constitute the initial and swift defensive barrier against viral infections [14]. The dysregulation of IFN-α has been observed in elderly COVID-19 patients, especially in severe cases, where IFN-α levels correlate with diminished counts of CD4 + and CD8 + T cells [15]. Therefore, measuring these mediators could be valuable in predicting the severity of the disease [11, 16]. Additional evidence indicates an association between parameters of systemic immune activation and serum bacterial DNA levels [17]. Indeed, a substantial body of evidence has demonstrated the presence of bacterial DNA in the bloodstream even in the absence of overt disease. Changes in the levels of this DNA as well as in the microbial community composition have been observed in aging, age-related diseases, cancer, cardiovascular, and autoimmune inflammatory diseases [18–22]. Emerging evidence is currently available regarding the correlation between circulating bacterial DNA levels in the blood and COVID-19 [23, 24]. Searching the mechanism of viral-induced susceptibility to secondary bacterial infection, Hanada and co-workers suggested a complex mechanism involving the interaction between the local and systemic immune response and the dysbiosis in the respiratory and gastrointestinal tract microbiota, which in turn may enhance the proliferation of potentially pathogenic bacterial species [25]. In this context, the detection of respiratory pathogens, including *Staphylococcus aureus, Pseudomonas aeruginosa,* and* Klebsiella pneumoniae*, in the blood from COVID-19 patients lead researchers to hypothesize that blood circulating bacterial DNA originates from secondary pneumonia or from the translocation of colonizing microbiota along the injured alveolar/epithelial surface of the lungs [24]. Furthermore, the findings of increased levels of (1 → 3)-β-d-glucan (BG), a biomarker of gut permeability, also suggest that the loss of gut barrier function may be a mechanism that contributes to the presence of bacterial toxin and DNA in the blood of patients with severe COVID-19 [23].

Since bacterial DNAemia represents an important candidate biomarker helpful for the prognosis and/or prediction of metabolic and clinical pathologic conditions, we quantified the bacterial DNA present in the bloodstream of 149 subjects of hospitalized elderly individuals affected by COVID-19 to identify its possible significant correlation with age-specific markers relevant for SARS-CoV-2 infection.

## Results

### Baseline characteristics of COVID-19 patients

The clinical characteristics of COVID-19 patients, divided into two groups based on the outcome of hospital stay, are summarized in Table 1.

**Table 1:** Baseline clinical patient characteristics

	**Discharged *n* = 102**	**Deceased *n* = 47**	***p*-value**
Age, mean ± SEM	84.00 ± 0.65	89.20 ± 0.97	** < 0.001**
Male sex, n (%)	38 (37.3%)	18 (38.3%)	0.907
Hypertension, n (%)	73 (73.0%)	34 (73.9%)	0.908
Diabetes, n (%)	27 (27.0%)	9 (19.6%)	0.333
COPD, n (%)	11 (11.0%)	11 (23.9%)	**0.043**
IHD, n (%)	11 (11.0%)	8 (17.4%)	0.286
AF, n (%)	26 (26.0%)	17 (37.0%)	0.177
Dementia, n (%)	31 (31.0%)	14 (30.4%)	0.945
CKD, n (%)	22 (21.5%)	17 (36.2%)	0.072
Respiratory insufficiency, n (%)	64 (62.7%)	43 (91.4%)	** < 0.001**
Fever, n (%)	48 (47.0%)	24 (51.5%)	0.725
Cough, n (%)	31 (30.4%)	6 (12.7%)	**0.020**
CFS n (%) 0–3	20 (19.6%)	(4.3%)	**0.035**
4–7	51 (50.0%)	24 (51.1%)	
8–9	29 (28.4%)	21 (44.7%)	
na	2 (2.0%)	0 (0.0%)	
CRP (mg/L), mean ± SEM^a^	5.15 ± 0.73	9.32 ± 1.05	** < 0.001**
Lymphocytes (10^3^/µL), mean ± SEM^a^	1.41 ± 0.10	1.03 ± 0.15	**0.004**
Neutrophils (10^3^/µL), mean ± SEM^a^	5.38 ± 0.47	8.73 ± 0.68	** < 0.001**
NLR, mean ± SEM^a^	4.87 ± 1.09	13.60 ± 1.56	** < 0.001**
Fibrinogen (mg/dL), mean ± SEM^a^	433 ± 17	460 ± 27	0.275
D-dimer (ng/mL), mean ± SEM^a^	1782 ± 371	2725 ± 601	0.130
Procalcitonin (ng/mL), mean ± SEM^a^	0.13 ± 0.35	2.29 ± 0.61	**0.001**
BB-DNA (ng/mL), mean ± SEM^a^	19.0 ± 8.7	49.7 ± 11.4	**0.015**

Chronic Obstructive Pulmonary Disease (COPD), Ischemic Heart Disease (IHD), Atrial Fibrillation (AF), Chronic Kidney Disease (CKD), C-Reactive Protein (CRP), Clinical Frailty Scale (CFS), Neutrophil to lymphocyte ratio (NLR), Not available (na), Standard Error of the Mean (SEM)

^a^ANCOVA analysis correcting for age, sex, and corticosteroid therapy

Patients deceased during the hospital stay were older than discharged patients and had an increased prevalence of Chronic Obstructive Pulmonary Disease (COPD), respiratory insufficiency, and cough. Moreover, deceased patients showed a significantly higher proportion of CFS (Clinical Frailty Scale) scores ≥ 8. Among laboratory parameters, deceased patients had increased CRP, neutrophil count, NLR and procalcitonin (*p*< 0.001) and decreased lymphocyte count (*p* < 0.01) compared to patients who survived. BB-DNA was higher in patients who deceased in-hospital, than patients who were discharged (*p* < 0.05).

### Cox regression analysis

A multivariate Cox regression analysis was performed to identify the main factors associated with in-hospital mortality in patients with COVID-19. The risk of mortality was greater in relation to BB-DNA (HR: 3.21; 95.0% CI: 1.34–7.68), age (HR: 1.11; 95.0% CI: 1.04–1.20), CRP (HR: 1.12; 95.0% CI: 1.04–1.20), neutrophil count (HR: 1.10; 95.0% CI: 1.02–1.19) and COPD (HR: 5.01; 95.0% CI: 1.67–14.97). In contrast, the risk of in-hospital death was lower in patients with higher lymphocyte count (HR: 0.40; 95.0% CI: 0.19–0.85) (Table 2).

**Table 2:** Multivariable Cox regression analysis of associated factors with in-hospital mortality in COVID-19 patients

	**HR**	**95% CI**	***p*-value**
BB-DNA	3.21	1.34–7.68	**0.008**
Age	1.11	1.04–1.20	**0.004**
Sex	1.89	0.83–4.32	0.128
CRP	1.12	1.04–1.20	**0.002**
Lymphocytes	0.40	0.19–0.85	**0.017**
Neutrophils	1.10	1.02–1.19	**0.009**
Diabetes	2.27	0.83–6.17	0.107
Hypertension	0.75	0.27–2.01	0.562
COPD	5.01	1.67–14.97	**0.004**
IHD	1.47	0.52–4.11	0.458
CKD	0.74	0.27–2.07	0.571
Dementia	0.55	0.21–1.38	0.202
AF	2.12	0.96–4.70	0.062
Ictus	1.90	0.66–5.48	0.233
CFS	1.17	0.95–1.44	0.134
Corticosteroid treatment	0.41	0.12–1.43	0.163

C-Reactive Protein (CRP), Chronic Obstructive Pulmonary Disease (COPD), Ischemic Heart Disease (IHD), Atrial Fibrillation (AF); Chronic Kidney Disease (CKD), Clinical Frailty Scale (CFS), Confidence Interval (CI)

Two additional multivariable Cox regression analyses have been conducted, with NLR or PLR serving as the independent variable. The risk of mortality was increased also in relation to PLR (HR:1.002; 95.0% CI: 1.001–1.004) and NLR (HR: 1.080; 95.0% CI: 1.044–1.118) (Table S1, Table S2).

**Fig. 1:**
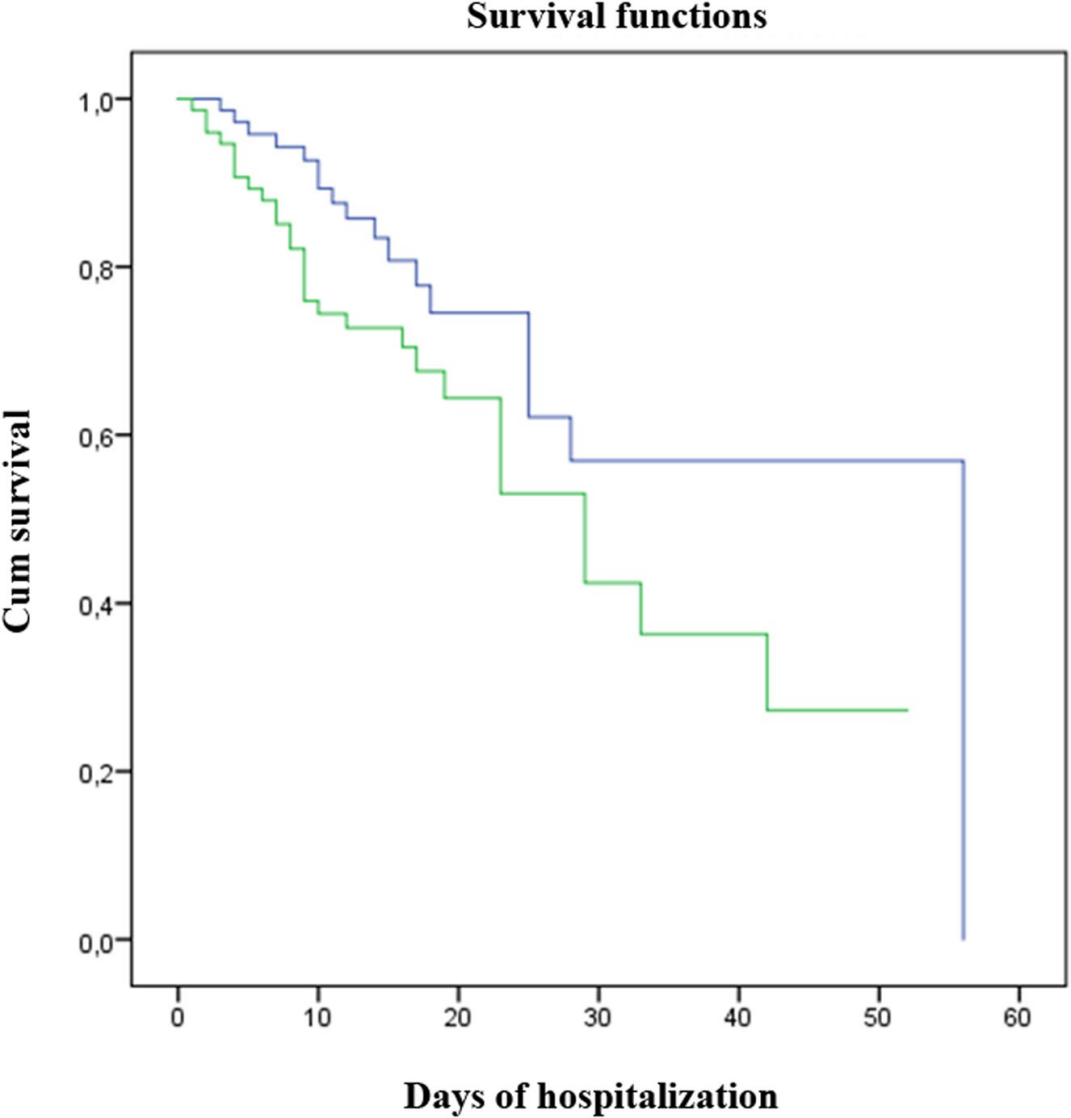
Kaplan–Meier survival estimates according to BB-DNA percentiles in old COVID-19 patients. Kaplan–Meier plots comparing in-hospital mortality between patients with BB-DNA < 9.62 ng/mL (blue line) and patients with BB-DNA ≥ 9.62 ng/mL (green line). Log Rank Χ^2^ = 3.929 *p* = 0.047

Kaplan–Meier analysis for the survival estimation over time confirmed a significantly lower survival rate in patients with the BB-DNA ≥ 9.62 ng/mL (*p* < 0.05; Fig. 1).

### Association between BB-DNA levels and plasma cytokine levels in old COVID-19 patients

**Fig. 2:**
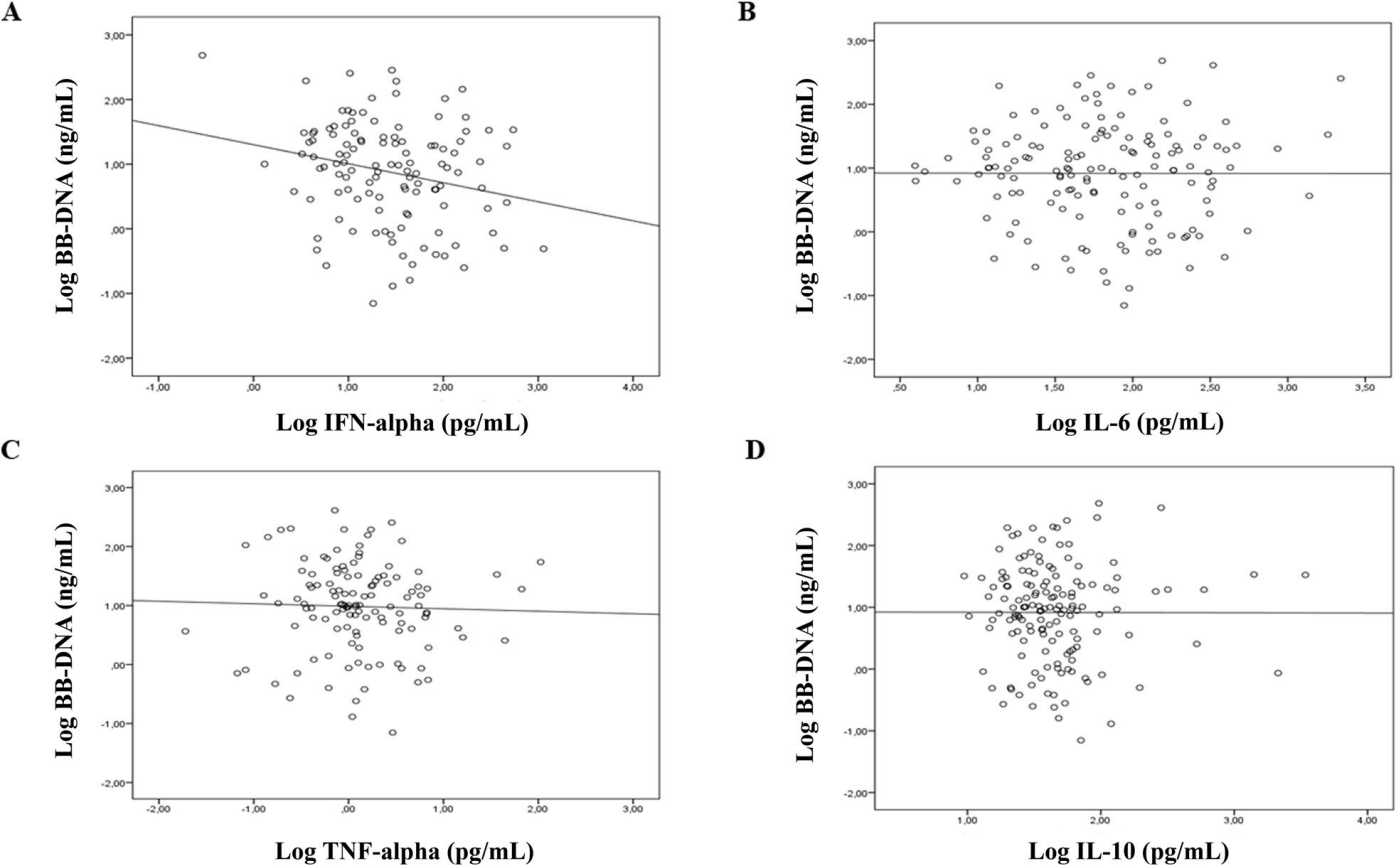
Linear regression between BB-DNA loads and plasma cytokine levels in old COVID-19 patients. A negative association between IFN-alpha and BB-DNA (Beta = -0.222, *p* = 0.019), after adjusting for age, sex, corticosteroid therapy and clinical frailty scale was found (**A**). No significant association were observed between BB-DNA and IL-6 (Beta = 0.001, *p* = 0.99), TNF-alpha (Beta = -0.045, *p* = 0.64). and IL-10 (Beta = -0.013, *p* = 0.87) (**B**, **C**, **D**). All data were log-transformed

Linear regression was used to evaluate the association between BB-DNA and plasma cytokine levels. BB-DNA was negatively associated with IFN-α (Beta = -0.222, *p* = 0.019) after adjusting for age, gender, corticosteroid therapy and clinical frailty scale (Fig. 2A). No significant association was found between BB-DNA and IL-6 (Beta = -0.001, *p* = 0.99) TNF-α (Beta = -0.045, *p* = 0.64) and IL-10 (Beta = -0.013, *p* = 0.87) (Fig. 2B, C and D).

### Association between BB-DNA levels and lymphocyte and neutrophil count, CRP, D-dimer and procalcitonin levels in old COVID-19 patients

**Fig. 3:**
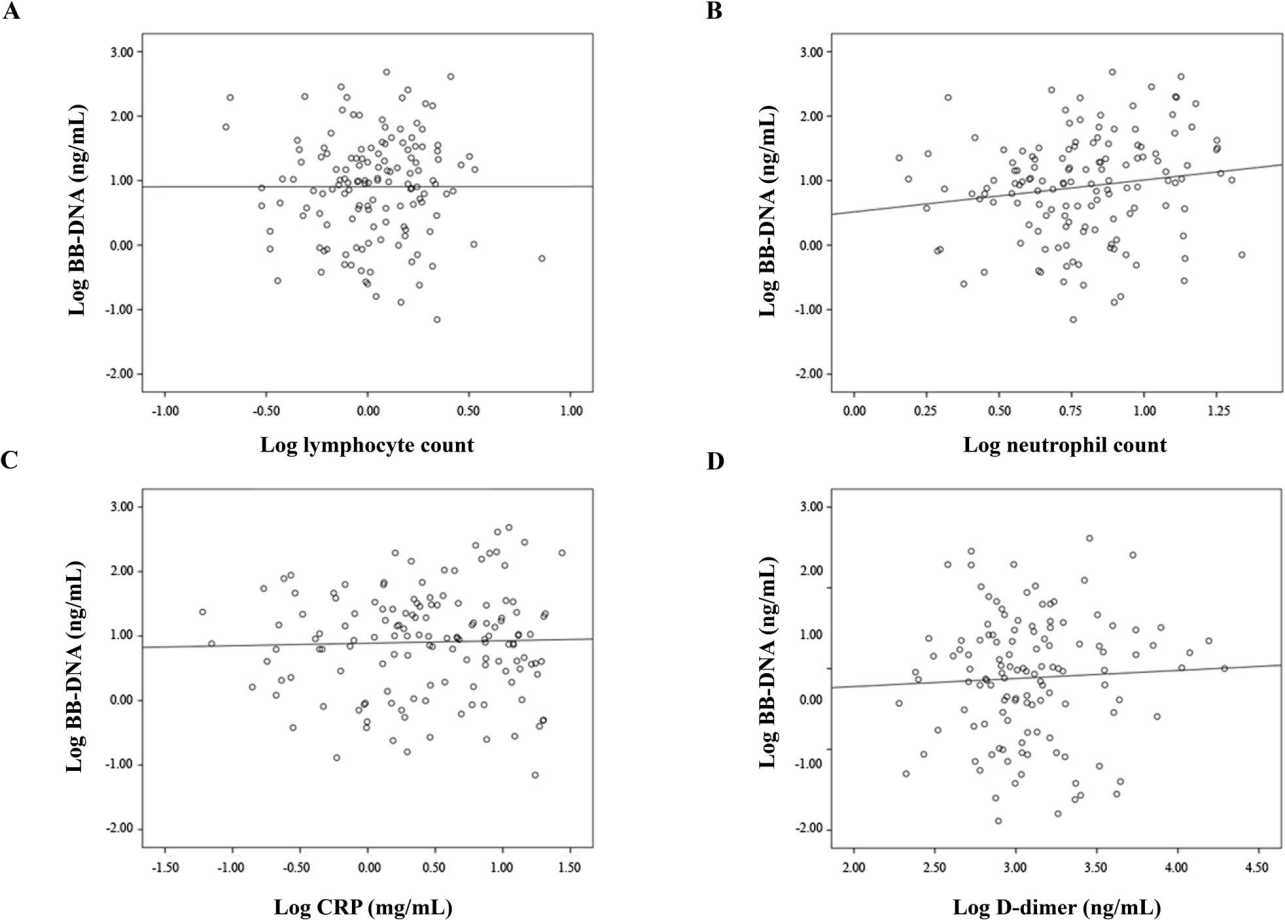
Linear regression between BB-DNA loads and lymphocyte and neutrophil count, CRP, and D-dimer levels in old COVID-19 patients. A positive association between neutrophil count and BB-DNA (Beta = 0.186, *p* = 0.045), after adjusting for age, sex, corticosteroid therapy and clinical frailty scale was found (**B**). No significant association were observed between BB-DNA and lymphocyte count (Beta = -0.020, *p* = 0.82), CRP (Beta = 0.042, *p* = 0.64) and D-dimer (Beta = 0.060, *p* = 0.52) (**A**, **C**, **D**). All data were log-transformed

A positive association between BB-DNA and neutrophil count (Beta = 0.186, *p* = 0.045) was found using linear regression after adjusting for age, gender, corticosteroid therapy and clinical frailty scale (Fig. 3B). No significant association was observed between BB-DNA and lymphocyte count (Beta = -0.020, *p* = 0.82), CRP (Beta = 0.042, *p* = 0.64) and D-dimer (Beta = 0.060, *p* = 0.52) (Fig. 3A, C, D). No significant association emerged between BB-DNA and procalcitonin serum levels (Beta = -0.051, *p* = 0.59) (data not shown).

### Multivariable logistic analysis between BB-DNA and comorbidities in COVID-19 patients

Table 3 shows the results of the multivariable logistic analysis to investigate BB-DNA as a possible predictor for some comorbidities. BB-DNA was independently associated with diabetes (OR: 1.005, 95% CI: 1.000–1.010, *p* = 0.040) after adjusting for age, gender, corticosteroid therapy and clinical frailty scale. BB-DNA was not associated with hypertension, ictus, COPD, ischemic heart disease, atrial fibrillation, dementia, or chronic kidney disease.

**Table 3:** Association among BB-DNA and comorbidities in adjusted multinomial logistic regression

Diseases	OR (95% CI) Model 1	*p*-value	OR (95% CI) Model 2	*p*-value
Diabetes	1.005(1.000–1.010)	**0.037**	1.005(1.000–1.010)	**0.040**
Hypertension	1.002(0.996–1.008)	0.528	1.002 (0.996–1.008)	0.542
Ictus	1.003(0.997–1.009)	0.335	1.003 (0.997–1.009)	0.375
COPD	0.998(0.991–1.006)	0.631	0.998 (0.991–1.005)	0.619
IHD	0.998(0.990–1.006)	0.665	0.998 (0.991–1.006)	0.694
AF	1.002(0.998–1.007)	0.322	1.002(0.997–1.007)	0.367
Dementia	1.000(0.995–1.005)	0.950	1.000(0.995–1.005)	0.953
CKD	1.001(0.996–1.006)	0.796	1.001(0.996–1.006)	0.753

Model 1 was adjusted for age, sex. Model 2 was adjusted for age, sex, corticosteroid therapy, and clinical frailty scale

Chronic Obstructive Pulmonary Disease (COPD), Ischemic Heart Disease (IHD), Atrial Fibrillation (AF), Chronic Kidney Disease (CKD), Confidence Interval (CI), Odds Ratio (OR)

## Discussion

Although early evidence described COVID-19 as a purely respiratory disease, several emerging clues demonstrate that it is a complex disorder of multi-organ dysfunction. It is still unclear why many individuals infected with SARS-CoV-2 are asymptomatic or develop a mild illness with symptoms such as fever, fatigue, dry cough, and headache [26, 27]. Similarly, a few clinical, comorbidities, demographics, and social factors have been progressively described that may explain the need for hospitalization of some individuals or the rapid progression of their symptoms to severe pneumonia/acute respiratory distress syndrome, requiring mechanical ventilation as well as the death of some [3, 28]. Older individuals were significantly impacted by COVID-19 due to the physiological changes associated with aging, decreased immune function, and multimorbidity that make them more susceptible to the infection itself and more likely to suffer severely from disease and serious complications [29].

Starting from the notion that a relationship between BB-DNA and pathological states exists, we hypothesized that different levels of BB-DNA in COVID-19-affected subjects may participate in the exacerbation of pathology and, thus, contribute as a cause of death. Our study finds that deceased patients had higher levels of BB-DNA than discharged patients and values of BB-DNA ≥ 9.62 ng/mL were associated with an increased mortality risk in hospitalized older patients, thus, highlighting that COVID-19 severity may be influenced by circulating bacterial DNA. We have previously reported the association of BB-DNA with different phenotypic traits, including free fatty acids levels, leukocyte count, insulin, and glucose levels, in subjects recruited from the general population aged 35 to 75 years old, with plasma BDNF levels in Alzheimer’s affected patients, and with plasma IL-10 and TNF-α levels in mild cognitive impairment subjects [18, 21, 22]. These observations, therefore, confirm and reinforce the assumption that BB-DNA could be an effective biomarker of human health and predictor of a disease prognosis. Although other authors have reported the association between BB-DNA and mortality in patients with COVID-19, our study is the first to demonstrate this in older subjects in which several age-related parameters were characterized [24]. We are confident in excluding that the presence of the bacterial DNA is attributable to hospital-acquired infections, since blood samples used for BB-DNA determination were collected at the beginning of hospitalization. Moreover, a series of data, concerning co-infections in patients with COVID-19, reported relatively low rates of co-infections between SARS-CoV-2 and other respiratory pathogens. In line with this, Garcia Vidal et al. underline that, if in the early phase of the COVID-19 pandemic the hospital protocol recommended antibiotic treatment for all patients, subsequently the observations led to reconsider this approach and to provide for administration only in situations of diagnosed/confirmed bacterial infections [30, 31]. Our study lacks the identification of SARS-CoV-2 variants, therefore no association of BB-DNA increases with specific SARS-CoV-2 variants has been evaluated. However, among the 149 patients, 113 were admitted during the period from November 2020 to June 2021 when the B.1.1.7 Alpha variant, associated with increased disease severity, prevailed in Italy [32–34]. The remaining 36 patients were hospitalized from March to October 2020, and it can be assumed that the dominant variant in our cohort is the SARS-CoV-2 lineage B.1.1.7. However, no significant differences in BB-DNA levels between the two groups were observed (16.3 ± 10.6 vs 35.7 ± 8.9, *p* > 0.05).

The associations we found between the mortality risk and increased age, higher neutrophil count, or COPD confirm literature data which consistently reports these factors as significant contributors to in-hospital mortality among COVID-19 patients [35, 36]. Additionally, low-levels of IFN-α have been associated with progression to severe COVID-19 [37, 38]. Therefore, the association between high neutrophils, low IFN-α levels, and high BB-DNA levels, emerged from our study, contributes to enhancing the identification of most risk factors of severe or fatal disease courses of the infection, thus helping its understanding and, therefore, treatment.

Interestingly, our study found an association between BB-DNA levels and diabetes. Patients with diabetes are characterized by dysbiosis and increased permeability of the intestinal barrier, which could be responsible for an increase in BB-DNA [39]. Furthermore, metagenomic analysis of nasopharyngeal microbiome from COVID-19 diabetic patients shows a significant increase in pathogens and secondary infection-causing bacteria compared with non-diabetic patients, thereby exacerbating the disease progression [40]. Conversely, we found no association between BB-DNA with other pre‐existing comorbidities such as hypertension, stroke, COPD, or ischemic heart disease. Very few studies in the literature measure BB-DNA levels in age-related diseases, and an increase in circulating bacterial DNA has been observed in cardiovascular (CVD) patients compared with healthy individuals and associated with cardiovascular events in peritoneal dialysis patients [41, 42]. However, the lack of association between BB-DNA and CVD may depend on the small sample size and differences in the clinical characteristics of the patients studied. We previously reported that high levels of BB-DNA were associated in healthy older subjects with increased insulin and glucose levels [18, 21]. Therefore, the correlation we observed between BB-DNA and diabetes in hospitalized older COVID-19 patients not only led to including both as prognostic markers in the assessment of severe COVID-19 disease but also to consider this association effective since it was detected in both physiological and pathological conditions.

The origin of bacterial DNA in blood is still subject to discussion. However, if we consider the various evidence discussed so far and correlate them to the data reported in the literature, it is plausible to retain that BB-DNA originates from translocation of bacteria into bloodstream, since SARS-CoV-2 infection alters the microbiota (dysbiosis) from different body districts (gut, skin, lung, respiratory tract) and impairs tissue barrier function [43–45]. Confirming this, as we discussed in Giacconi et al., high levels of insulin, found associated with BB-DNA, have been implicated in gut permeability and impaired fasting glucose subjects showing higher markers of gut permeability than controls [46]. Similarly, gut, and oral microbiota dysbiosis and different microbiome compartmentalization have been observed in Type 2 diabetes subjects [47].

This study has some limitations, such as the small sample size of our cohort, the unbalanced distribution of sex, the evaluation of a single outcome, i.e.in-hospital mortality, the lack of assessment of disease severity by means some clinical scales such as the sepsis-related organ failure assessment (SOFA), and the score for pneumonia severity (CURB-65) scales, the lack of therapy evaluation carried out during admission, which might affect patients’ evolution and the lack of a metagenomic analysis for blood microbiome signature. Importantly, recent evidence demonstrated that gut dysbiosis in COVID-19 patients is associated with secondary bloodstream infections by gut opportunistic pathogenic bacteria [48]. Therefore, the measurements of BB-DNA in COVID-19 patients might represent a useful biomarker of bacterial secondary infections and help clinicians in the early identification of patients at high risk of more severe outcomes. Future studies should also evaluate if treatments (e.g., with micronutrients), addressed to reduce the endothelial dysfunction and mucosal barrier damage, could reduce BB-DNA levels, and improve patient prognosis.

## Conclusions

Our results reinforce the idea that increments of BB-DNA, likely due to increased endothelial dysfunction and mucosal permeability with consequent bacterial translocation in old COVID-19 patients, aggravated by the presence of diabetes, can be relevant to the severity of illness and might increase the risk of in-hospital mortality.

## Materials and methods

Demographic and clinical characteristics of study participants.

This study included 149 older patients with COVID-19 (age range 65–99 years, 93 females and 56 males) who were recruited, between March 2020 and June 2021, as part of Report-Age COVID-19 project, an observational study conducted at the Italian National Center on Aging (IRCCS INRCA). All patients included in the study had not received vaccination against SARS-CoV-2. Corticosteroid therapy was administered to 84% of the patients.

The study was approved by the Ethics Committee of IRCCS INRCA (number CE-INRCA-20008) and registered in the ClinicalTrials.gov database (reference number NCT04348396). All patients signed informed consent. The study protocol was performed in alignment with local and international guidelines and regulations, and the research has been conducted in accordance with the Declaration of Helsinki.

Nasopharyngeal and throat swab samples were obtained at admission from all patients who were tested using the real-time polymerase chain reaction (RT-PCR) assay to identify SARS-CoV-2 infection.

Clinical data included symptoms and signs of infection, such as fever, cough, dyspnea, diarrhea, nausea, and vomiting. Frailty was graded according to the Rockwood Clinical Frailty Scale (CFS) [49]. Comorbidities were assessed, including hypertension, diabetes mellitus, chronic obstructive pulmonary disease, ischemic heart disease, atrial fibrillation, dementia, and chronic kidney disease.

Laboratory parameters at admission, including complete blood count, serum concentrations of C-reactive protein (CRP), fibrinogen, D-dimer, were tested in the IRCCS INRCA laboratory using standardized and certified procedures.

### SARS-CoV-2 PCR method

The presence of SARS-CoV-2 RNA in plasma was determined by examining 10 μl of nucleic acids extracted from plasma using the Real Quality RQ-SARS-CoV-2 real-time PCR assay (AB Analitica). This assay specifically targeted the RdRp gene (which encodes the RNA-dependent RNA polymerase) and the S gene (which encodes the spike protein). An endogenous control, targeting the human RNAse P gene, was incorporated into the same assay as an internal control to verify the accuracy of sampling and nucleic acids extraction. The assay was conducted according to the manufacturer's specified conditions on an Agilent Aria Mx Real-Time PCR instrument. A plasma sample was classified as positive for SARS-CoV-2 RNAemia only if both viral genes were identified with a cycle threshold (CT) below 40.

### Blood Bacterial DNA (BB-DNA) quantification

DNA was extracted from 300 µL of whole blood samples using the QIAamp DNA Blood mini kit (Qiagen GmbH, Hilden, Germany) according to the manufacturer’s instructions. Blood Bacterial DNA (BB-DNA) quantification was carried out by the amplification of the 16S rRNA gene as previously reported [18, 21, 22]. Briefly, specific universal primers targeting the V3–V4 hypervariable region of the bacterial 16S rDNA were used in real-time qPCR reactions. The PCR mixture (20 µL) consisted of 20 ng of DNA, SensiFAST SYBR Hi-ROX Mix 1 × (Bioline, London, UK), and 0.4 µM of the following primers: For 5′-TCCTACGGGAGGCAGCAGT-3′ and Rev 5′-GGACTACCAGGGTATCTAATCCTGTT-3′. The thermal profile used for the reaction included a heat activation of the enzyme at 95 °C for 2 min, followed by 40 cycles of denaturation at 95 °C for 15 s and annealing/extension at 60 °C for 60 s, followed by melt analysis ramping at 60–95 °C. All measurements were taken in the log phase amplification. Standard curves obtained using a tenfold dilution series of bacterial DNA standards (Femto bacterial DNA quantification kit, Zymo Research) ranging from 0.0002 to 2 picogrammes were routinely run with each sample set and compared with previous standard curves to check for consistency between runs. Amplicon quality was ascertained by the melting curves. Amplifications of samples and standard dilutions were performed in triplicate on the QuantStudio 3 Real-Time PCR System (ThermoFisher Scientific). BB-DNA levels were expressed as nanograms per millilitre of whole blood and were calculated by normalizing the absolute quantities of BB-DNA of each sample to their dilution factors and to the volume of starting blood used for the extraction.

A series of controls were performed to exclude artefacts from sample manipulation, reagent contamination, and non-specific amplifications. The primers were checked for possible cross-hybridization with genes from eukaryotic and mitochondrial genomes using the similarity search tool BLAST. The BLAST results showed no hits, thus confirming the specificity of primers for the bacterial 16S rRNA as previously reported [50]. Separate working areas for real-time PCR mix preparation, template addition, and performing the PCRs were used. All experimental procedures were performed under a laminar flow hood using dedicated pipettes, filter-sealed tips, and plasticware guaranteed to be DNA-free. Negative controls, in which ultrapure water instead of DNA was added, were also run in each plate.

### Cytokine determination

Serum IFN-α, IL-6, TNF-α, and IL-10 were measured by using the high-sensitivity ProQuantum qPCR immunoassays (ThermoFisher Scientific) on the Aria Mix real-time PCR system (Agilent) following the manufacturer protocol. Each sample was assayed in duplicate. The correlation between IL-6 levels measured by the ProQuantum qPCR immunoassays method and those by the Elecsys IL-6 ELISA kit (Roche, Milan, Italy) (range from 1.98 to 4372 pg/mL) was assayed. Cytokine values were positively correlated with Spearman rho = 0.602, *p* < 0.0001 (Figure S1).

### Statistical analysis

Log transformation of the variables was carried out, if they were not normally distributed, as assessed by the Kolmogorov–Smirnov test. Continuous variables were reported as mean and standard deviation, and categorical variables were described as frequency and percentage. The median and interquartile range (IQR) were described by applying the Kruskal–Wallis test.

Differences between groups were analyzed by ANOVA (after correction for age and gender) for continuous variables and Pearson’s χ2 test for categorical variables.

Kaplan–Meier and Cox regression analyses investigated the relationship between BB-DNA and mortality. The main factors included in the Cox regression model were BB-DNA, age, sex, CRP, lymphocyte and neutrophil count, comorbidities, and clinical frailty scale. The association between BB-DNA and inflammatory or laboratory parameters was analyzed by linear regression. Multinomial logistic regression was used to identify risk factors for comorbidities in COVID-19 patients.

Statistical significance was set to 2-sided *P* < 0.05. Statistical analyses were performed using SPSS (Version 27.0.1.0, IBM).

### Supplementary Information

**Additional file 1.**

## Data Availability

All data generated or analysed during the current study are available from Robertina Giacconi upon reasonable request.
