# Supplementary material for: Blood circulating bacterial DNA in hospitalized old COVID-19 patients
Source: Immun Ageing. 2023 Dec 18;20:76. doi: 10.1186/s12979-023-00401-4 (PMC10726536; doi:10.1186/s12979-023-00401-4)
Supplement: Supplementary file 1 — Additional file 1. [file 12979_2023_401_MOESM1_ESM.pdf]

SUPPLEMENTARY MATERIAL

Table S1. Multivariable Cox regression analysis of associated factors with in-hospital mortality in COVID-19 patients including NLR as independent variable.

	**HR**	**95% CI**	**p-value**
BB-DNA	1.006	1.002-1.010	0.002
Age	1.079	0.999-1.164	0.053
Sex	0.645	0.293-1.420	0.277
CRP	1.105	1.034-1.182	0.003
NLR	1.080	1.044-1.118	<0.0001
Diabetes	0.921	0.309-2.741	0.882
Hypertension	1.333	0.477-3.723	0.584
COPD	6.954	2.269-21.315	0.001
IHD	1.005	0.340-2.970	0.993
CKD	0.521	0.200-1.361	0.183
Dementia	0.823	0.309-2.192	0.696
AF	2.812	1.244-6.355	0.013
Ictus	0.652	0.211-2.017	0.458
CFS	1.167	0.934-1.458	0.175
Corticosteroid treatment	0.528	0.154-1.805	0.309

C-Reactive Protein (CRP), Chronic Obstructive Pulmonary Disease (COPD); Ischemic Heart Disease (IHD); Atrial Fibrillation (AF); Chronic Kidney Disease (CKD); Clinical Frailty Scale (CFS) Confidence Interval (CI) NLR (neutrophil to lymphocyte ratio)

Table S2. Multivariable Cox regression analysis of associated factors with in-hospital mortality in COVID-19 patients including PLR as independent variable.

	**HR**	**95% CI**	**p-value**
BB-DNA	1.004	1.000-1.007	0.032
Age	1.102	1.022-1.189	0.012
Sex	0.967	0.452-2.073	0.932
CRP	1.100	1.031-1.174	0.004
PLR	1.002	1.001-1.004	0.007
Diabetes	1.380	0.525-3.626	0.514
Hypertension	1.213	0.451-3.264	0.752
COPD	3.746	1.364-10.286	0.010
IHD	1.391	0.516-3.752	0.514
CKD	0.731	0.299-1.783	0.490
Dementia	0.734	0.290-1.861	0.515
AF	2.465	1.120-5.426	0.025
Ictus	1.181	0.410-3.402	0.758
CFS	1.079	0.881-1.322	0.464
Corticosteroid treatment	0.746	0.228-2.446	0.629

C-Reactive Protein (CRP), Chronic Obstructive Pulmonary Disease (COPD); Ischemic Heart Disease (IHD); Atrial Fibrillation (AF); Chronic Kidney Disease (CKD); Clinical Frailty Scale (CFS); Confidence Interval (CI); PLR (platelet to lymphocyte ratio)

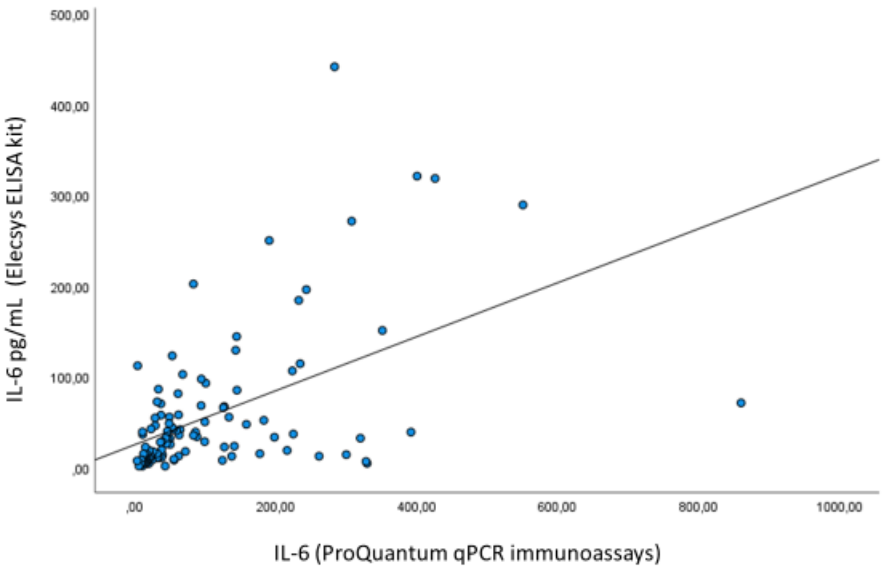

Figure S1. Spearman correlation between IL-6 serum levels assessed using the Elecsys ELISA kit and ProQuantum qPCR immunoassays.

Serum IL-6 levels were determined using two distinct assays: ProQuantum qPCR immunoassays (ThermoFisher Scientific) and the Elecsys IL-6 ELISA kit (Roche, Milan, Italy), which featured a detection range of 1.98-4372 pg/mL and a sensitivity of 1.5 pg/mL. Cytokine values were positively correlated with Spearman rho=0.602, $p<0.0001$
